# Sidewalk Hazard Detection Using a Variational Autoencoder and One-Class SVM

**DOI:** 10.3390/s26030769

**Published:** 2026-01-23

**Authors:** Edgar R. Guzman, Robert D. Howe

**Affiliations:** Harvard John A. Paulson School of Engineering and Applied Sciences, Harvard University, Cambridge, MA 02138, USA; howe@seas.harvard.edu

**Keywords:** hazard, anomaly, computer vision, navigation

## Abstract

The unpredictable nature of outdoor settings introduces numerous safety concerns, making hazard detection crucial for safe navigation. To address this issue, this paper proposes a sidewalk hazard detection system that combines a Variational Autoencoder (VAE) with a One-Class Support Vector Machine (OCSVM), using a wearable RGB camera as the primary sensing modality to enable low-cost, portable deployment and provide visual detail for detecting surface irregularities and unexpected objects. The VAE is trained exclusively on clean, obstruction-free sidewalk data to learn normal appearance patterns. At inference time, the reconstruction error produced by the VAE is used to identify spatial anomalies within each frame. These flagged anomalies are passed to an OCSVM, which determines whether they constitute a non-hazardous anomaly or a true hazardous anomaly that may impede navigation. To support this approach, we introduce a custom dataset consisting of over 20,000 training images of normal sidewalk scenes and 8000 testing frames containing both hazardous and non-hazardous anomalies. Experimental results demonstrate that the proposed VAE + OCSVM model achieves an AUC of 0.92 and an F1 score of 0.85, outperforming baseline anomaly detection models for outdoor sidewalk navigation. These findings indicate that the hybrid method offers a robust solution for sidewalk hazard detection in real-world outdoor environments.

## 1. Introduction

Vision is an essential sense that helps perceive and interpret the environment, allowing navigation, decision-making, and interaction with the surroundings. As humans, we rely on vision to connect with our environment to perform fast and accurate tasks in complex settings. However, navigating through the world can be challenging for many, including the elderly, the visually impaired, and even robots. Thus, research in safety navigation has made great progress in the past decade, creating intuitive systems capable of generating trajectory plans and preventing collisions [1,2]. Most of these systems have been implemented and evaluated as navigation aids that guide users around potential hazards. Yet, many lack robustness in accurately detecting hazards and can fail in situations where such hazards are not properly identified. For widespread adoption, safety systems must integrate reliable hazard detection.

A promising approach to gathering information about the environment involves the use of inexpensive cameras. These sensors are capable of detecting objects within each frame [3], potentially indicating the presence of a hazard. However, most vision-based navigation systems rely on supervised learning, which demands large amounts of manually labeled data, which is costly and difficult to obtain, especially for hazardous scenarios. Moreover, supervised models struggle to generalize to unseen hazards due to the limited diversity of labeled examples [4].

To overcome these limitations, unsupervised and self-supervised learning models have emerged as a powerful method to leverage unlabeled data by learning meaningful representations without the need for exhaustive manual annotation [5,6]. These models have shown strong performance in anomaly detection across domains such as surveillance, transportation, and infrastructure monitoring [7,8,9]. In safety navigation, anomaly detection has also been applied to detect irregularities in robot and vehicle trajectories [10,11,12].

While vision-based machine learning approaches have proven to enhance the detection of anomalies in many fields, their applications to safety navigation for the elderly or visually impaired have not advanced as rapidly. To address this gap, we present a hazard detection system designed for sidewalk navigation, leveraging a Variational Autoencoder (VAE) fused with a One-Class Support Vector Machine (OCSVM) to process RGB data. In addition, we introduce a novel egocentric navigation sidewalk dataset, specifically curated to capture real-world variations in pedestrian environments.

In the proposed approach, the VAE is trained solely on clean, hazard-free sidewalk images to learn a compact representation of normal scenes. During inference, the VAE reconstructs each input frame and computes a reconstruction error, which is used to identify regions that deviate from learned normal patterns, indicating potential anomalies. These anomaly-flagged compact representations are then passed to an OCSVM, which distinguishes between non-hazardous anomalies and true hazardous anomalies that may impede safe navigation. This two-stage design allows the system to detect hazards without requiring large annotated datasets and enables generalization to previously unseen hazard types.

The main contribution of this work is a hazard detection framework tailored for egocentric sidewalk navigation. While prior studies have explored the combination of Variational Autoencoders and One-Class SVMs for general anomaly detection, this work adapts and evaluates a VAE + OCSVM framework for egocentric sidewalk hazard detection, demonstrating its effectiveness in a real-world navigation setting. Our framework leverages a VAE for anomaly localization and an OCSVM for hazard discrimination without requiring large labeled hazard data, enabling strong generalization to unseen obstacles. In addition, we introduce a new egocentric sidewalk dataset designed for anomaly detection under natural outdoor conditions. The proposed system achieves strong performance metrics and offers a practical, sensor-efficient solution suitable for portable assistive devices and real-world deployment.

In the next section, we describe the related works in terms of anomaly detection and examine existing approaches for hazard detection in outdoor settings. Afterwards, we describe the system’s design and provide an overview. We then proceed to describe the methodology of our study, including the dataset utilized and the approaches employed across the VAE and the OCSVM. Subsequently, we present a section dedicated to the results, where we evaluate the effectiveness and accuracy of the VAE + OCSVM compared to other baseline models. The paper concludes with a discussion of the implications of our findings, addressing system-level considerations and exploring potential directions for future research in this domain.

## 2. Related Work

**Anomaly Detection:** Traditional anomaly detection techniques, such as Gaussian Mixture Models (GMMs) and Principal Component Analysis (PCA), model normal distributions and identify deviations as anomalies [13,14]. However, GMMs assume a Gaussian distribution, which may not generalize well to the diverse textures and lighting variations present in sidewalk environments. Another common approach for anomaly detection is the use of SVMs, as they can learn a boundary between recognized and non-recognized data [15,16,17]. However, SVMs struggle with high-dimensional data due to their high computational cost and extensive memory demands. Methods like Isolation Forest (IF) tend to perform poorly in high-dimensional latent spaces, as they rely on random partitioning rather than structured decision boundaries [18].

More recently, deep learning-based methods have emerged, demonstrating the ability to learn complex data distributions in a more flexible manner. In general, deep learning-based anomaly detection models can be categorized into discriminative models and generative models. Discriminative models focus on distinguishing normal data from anomalies by learning decision boundaries, such as Deep Support Vector Data Description (DeepSVDD) [19]. Deep SVDD has been used for anomaly detection but suffers from hypersphere collapse, where all normal data points are mapped to a single cluster. This issue arises when the training dataset lacks variation, leading to poor generalization in real-world navigation scenarios.

Generative models aim to learn the probability distribution of normal data, including methods like Real-valued Non-Volume Preserving transformation (RealNVP) [20] and Variational Autoencoders (VAEs) [21]. These generative methods can reconstruct input data, identifying anomalies based on low-likelihood estimates or high reconstruction errors, respectively. However, RealNVP requires careful adversarial training, making it challenging to stabilize, especially for real-time deployment in safety-critical navigation. Normalizing flows, while offering exact likelihood estimation, are computationally expensive and harder to scale to embedded or mobile platforms.

In parallel, recent advances in video anomaly detection have explored temporal modeling for anomaly recognition [22]. Methods such as transformer-based models, patch-level spatio-temporal relation prediction, and multi-scale spatio-temporal representation learning [23,24,25]. These methods demonstrate strong performance in surveillance-style anomaly detection by leveraging long-range temporal context and rich spatio-temporal features. However, they typically assume fixed camera viewpoints and require substantial computational cost, which limits their applicability to real-time egocentric navigation using wearable sensors.

While the combination of VAE and OCSVM has been explored in previous works for anomaly detection [26,27,28], its application to sidewalk hazard detection has not been studied. These approaches are designed for environments where anomalies occur within a fixed field of view. However, in safety navigation systems, the dynamic nature of pedestrian movement introduces unique challenges that remain underexplored.

**Hazard Detection for Sidewalk Navigation:** Recent advancements in assistive technology for the visually impaired and lower-limb assistive devices have integrated wearable RGB cameras to help users avoid hazardous situations, such as changes in terrain (e.g., staircases, ramps, curbs) [29,30,31]. Other research has explored hazard detection in traversal areas using a polarized RGB-D camera, leveraging polarized light reflection to identify puddles [32]. While effective in detecting water hazards and traversable areas, this method struggles with small obstacles due to depth filtering and dynamic programming limitations.

Another approach involves training a convolutional neural network (CNN) to detect terrain changes and identify hazardous potholes within a walking trajectory [33]. Its effectiveness is limited by the diversity of its training dataset and the number of hazard types it can recognize. To expand the range of detectable hazards, researchers have developed deep learning models, such as a RESNET-based architecture that classifies hazards into five categories: Curb Ramp, Missing Curb, Surface Problem, Obstruction, and Null [34]. Similarly, vision-language models for video anomaly detection, such as VisionGPT [35], utilize YOLO World to detect potentially hazardous objects in the environment. While these methods provide useful context for blind users, they lack the precision to classify all hazards, which is crucial for delivering real-time warnings.

A major limitation affecting these models is the availability and quality of sidewalk navigation datasets. Many existing datasets require images to be cropped from Google Street View, while others provide RGB images with a field of view primarily directed toward the horizon [36]. However, research indicates that pedestrians naturally focus their visual gaze forward rather than on the ground immediately in front of them, limiting their ability to detect subtle details on the walking surface [37]. Furthermore, studies suggest that when navigating familiar pathways, individuals tend to fixate more on stepping targets (spots on the ground), a strategy that supports safe foot placement [38]. This discrepancy highlights the need for datasets that better capture the pedestrian’s immediate walking area, ensuring safe navigation via hazard detection.

## 3. System Design

### 3.1. Overview

The Variational Autoencoder (VAE) offers a probabilistic latent space, enhancing robustness to anomalies that slightly deviate from the training data. Unlike other autoencoders [39,40], VAEs incorporate stochasticity through the reparameterization trick, enabling better generalization to variations in sidewalk textures and environmental conditions. This approach balances feature extraction, generalizability, and computational efficiency, making it well-suited for hazard detection in sidewalk navigation.

For hazard classification, the One-Class SVM (OCSVM) efficiently learns a flexible decision boundary within the latent space. OCSVM effectively distinguishes hazardous and non-hazardous anomalies while maintaining a lightweight computational cost [41]. Unlike contrastive learning approaches that infer anomalies indirectly through feature similarity or distance, OCSVM explicitly models the boundary of normal behavior, yielding more stable and interpretable anomaly decisions with significantly lower computational overhead.

**Figure 1:**
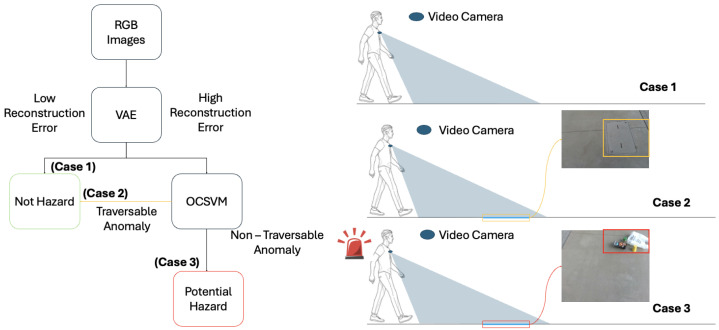
Overview diagram of the proposed approach with deployment cases.

In the proposed system, the user wears a video camera as they navigate sidewalks, where RGB frames are passed through a Variational Autoencoder (VAE) that has been trained on sidewalks without hazards. If the encode-decode error is high, the VAE has identified an anomaly, which could potentially be hazardous. In these cases, the VAE’s latent vector is then passed to an OCSVM. The OCSVM learns a decision boundary around latent representations of non-hazardous, traversable anomalies, enabling hazardous anomalies to be identified as outliers during inference. If a hazardous anomaly is detected, the system creates a bounding box over the detected anomaly, alerting the user to the location of the hazard. Figure 1 provides a high-level overview of the proposed system’s flow as well as a visualization of the application.

### 3.2. Data Collection

Normal sidewalk samples were collected using an RGB camera, capturing frames from traversable, obstruction-free sidewalks, including cement and brick surfaces. The camera was positioned at chest height and tilted approximately 60 degrees downward to focus on the sidewalk immediately in front of the user. During data collection, the user walked at a normal, steady walking speed representative of typical pedestrian navigation. To ensure the training data were diverse and generalizable, images were collected from several sidewalk locations that shared the same terrain type but differed in appearance due to variations in texture, lighting, and surroundings. Additionally, data augmentation was applied by adjusting brightness levels to simulate varying lighting conditions, including bright and cloudy days.

After normal data was collected, abnormal objects on various types of sidewalks were captured for testing purposes. Some of these anomalies were naturally occurring, like large cracks in the sidewalk, while others, such as litter, were artificially introduced to validate the concept.

All experiments were conducted using an Intel RealSense D435i RGB camera (Santa Clara, CA, USA). The camera provides RGB images at a resolution of 640 × 480 pixels with a field of view of 87° × 58°, operating at 30 frames per second. Data collection was performed using an HP Envy laptop running Ubuntu 18.04.6 LTS, which interfaced directly with the Intel RealSense D435i camera during outdoor recording sessions.

Model training was performed on the Harvard FAS Research Computing (FASRC) cluster. All model inference, evaluation, and runtime performance measurements were conducted on an Apple M2 processor using Metal Performance Shaders (Apple Inc., Cupertino, CA, USA). This setup reflects realistic deployment constraints for portable assistive navigation systems. The depth of infrared data from the camera was not used in this study.

### 3.3. Variational Autoencoder

**Figure 2:**
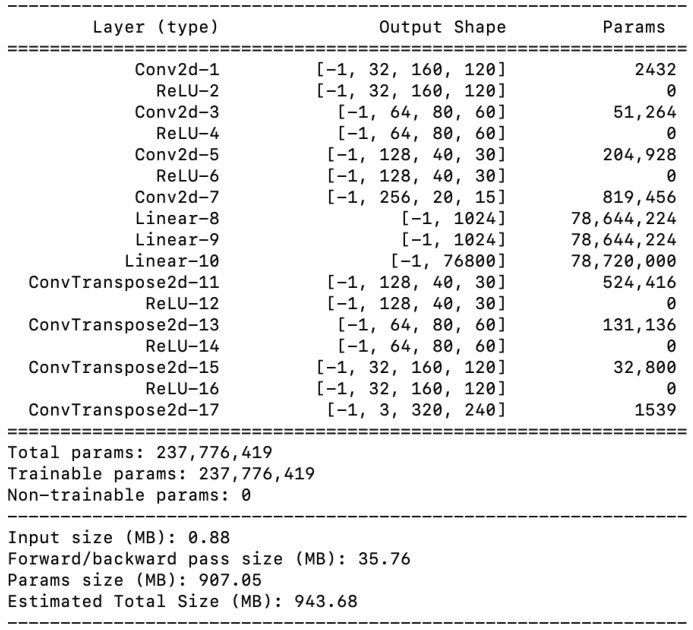
Variational Autoencoder architecture.

**Figure 3:**
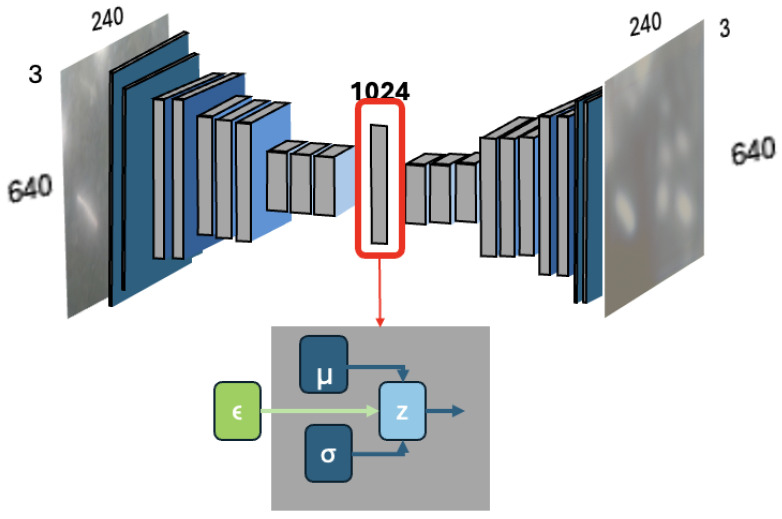
Variational Autoencoder latent space reparameterization trick. The blue arrows indicate the learned mean (μ) and standard deviation (σ) outputs of the encoder, while the green arrow represents the random noise term ϵ used for stochastic sampling of the latent variable *z*.

Variational Autoencoders (VAEs) are deep neural networks composed of an encoder, F(x), where *x* is the input. The encoder utilizes convolutional layers and ReLU activations (as shown in Figure 2) to transform input images into a low-dimensional feature space, producing a mean vector μ and a standard deviation vector σ as shown in Figure 3, which defines a Gaussian distribution N(μ,σ). A latent vector, *z*, is derived by sampling ϵ, a random noise variable, from a standard normal Gaussian distribution (ϵ∼N(0,1)) and then mapping it back to μ and σ such that z=μ+σ×ϵ. This process, known as the reparameterization trick, allows for stochastic sampling while keeping the model differentiable, enabling backpropagation during training. This method introduces stochasticity, which is advantageous compared to the deterministic output from traditional autoencoders because it allows the model to learn and generate a diverse set of latent representations. The decoder, $F^{\prime }\left(z\right)$, also built with convolutional layers and ReLU activations (as shown in Figure 2), then attempts to reconstruct the original image from the latent vector. For further details of VAE, see [42].

The VAE is trained to accurately capture the characteristics of the training data variability. In this paper, the training data consists of variations in normal sidewalk images. As a result, during deployment, the VAE will struggle to produce an appropriate latent vector when presented with images containing anomalies, leading to a high reconstruction error.

Unlike traditional autoencoders, a Variational Autoencoder (VAE) optimizes both the reconstruction accuracy and the regularization of the latent space. The overall loss function is defined as

(1) $$L=E_{q_{\phi }\left(z|x\right)}\left[\log p_{\theta }\left(x|z\right)\right]-\mathrm{KL}\left(q_{\phi }\left(z|x\right)\|p\left(z\right)\right)$$

where ϕ and θ represent the parameters of the encoder and decoder networks, respectively. The first term, $E_{q_{\phi }\left(z|x\right)}\left[\log p_{\theta }\left(x|z\right)\right]$, denotes the reconstruction probability, which measures how well the decoder can reconstruct the input data *x* from the latent variable *z*. The second term, $\mathrm{KL}(q_{\phi }\left(z|x\right)\|p\left(z\right))$, represents the Kullback–Leibler (KL) divergence, which quantifies the difference between the encoder’s learned distribution $q_{\phi }\left(z|x\right)$ and the prior distribution p(z). In this work, p(z) is modeled as a standard normal distribution, N(0,1), encouraging the latent space to remain continuous and well-structured.

By leveraging this loss formulation, the reconstruction probability is used to determine the presence of anomalies within each frame, following the approach in [42]. The threshold is determined empirically through a grid search, in which a range of values is evaluated to find the one that best separates normal and anomalous samples. If the reconstruction probability falls below this threshold, the frame is labeled as anomalous (−1); otherwise, it is considered normal (+1).

### 3.4. One Class SVM

The OCSVM is a useful tool for anomaly detection, capable of generating a decision boundary to separate data. However, it struggles when dealing with high-dimensional data, such as images. To address this, we utilize the latent space of a Variational Autoencoder (VAE), which compresses input images into a lower-dimensional 1024-element vector. By training an OCSVM on this latent representation, we establish a decision boundary to differentiate between non-hazardous anomalies and hazardous anomalies.

**Figure 4:**
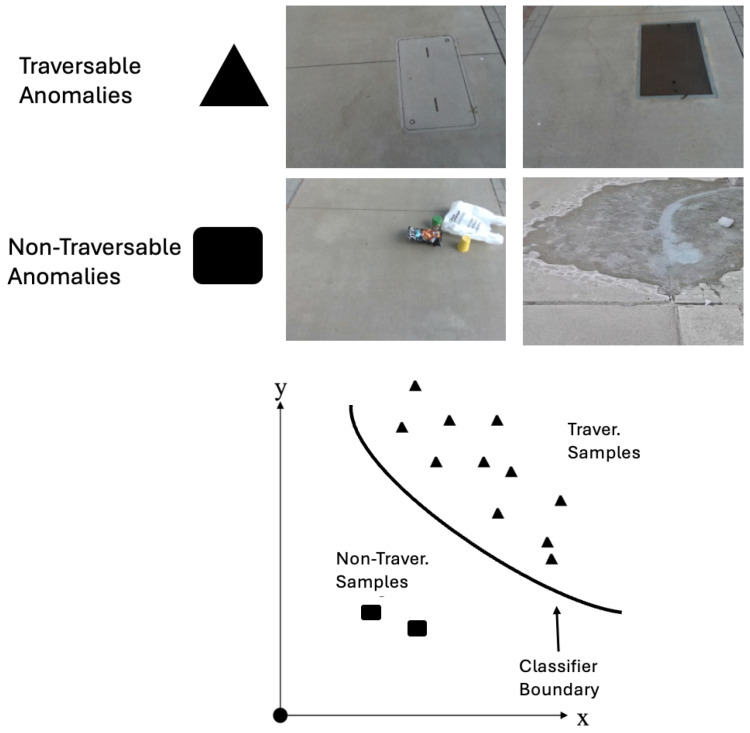
Support Vector Machine Class Distinction.

To ensure a proper decision boundary, the OCSVM is trained exclusively on data representing traversable non-hazardous anomalies, which refer to environmental variations that do not pose risks to navigation. In this work, non-hazardous anomalies are defined as visually irregular sidewalk elements that remain safely traversable and do not require changes in walking trajectory or gait under typical pedestrian navigation. Examples include water meter covers and manholes, which are anomalous in appearance but do not impede forward motion (Figure 4). The latent vectors are normalized to ensure that all features contribute equally during the model’s learning process.

Given the non-linear nature of the data distribution, a radial basis function (RBF) kernel with a scaling gamma (γ) is used to create the decision boundary. The RBF kernel effectively maps the data into a higher-dimensional space, enabling the OCSVM to better distinguish between traversable anomalies and non-traversable anomalies. As a result, the OCSVM decision function returns +1 for traversable non-hazardous anomalies and −1 for non-traversable hazardous anomalies, as shown in Equation (2).

(2) $$f\left(x\right)=sgn\left(\sum_{i=1}^{n}\alpha_{i}\cdot \exp(-\gamma ∥x-x_{i}{∥}^{2})-\mu \right)$$

where $\alpha_{i}$ are the Lagrange multipliers, which determine the contribution of each support vector in shaping the decision boundary. These values are optimized during training and are nonzero for the support vectors. γ is the scaling parameter for the RBF kernel, controlling the influence each training point has on the decision boundary. $∥x-x_{i}{∥}^{2}$ is the squared Euclidean distance between the input *x* and the learned support vectors $x_{i}$. μ is the bias term, a constant that adjusts the separation between traversable and non-traversable anomalies during training.

Since the OCSVM training dataset contains only non-hazardous, traversable anomalies, the decision boundary is learned solely from this class, allowing hazardous anomalies to be detected as outliers at inference time. Moreover, γ was defined as

(3) $$\gamma =\frac{1}{f\times \sigma }$$

where *f* represents the features in the latent vector, and σ is the mean variance computed across all features. This allows γ to adapt to the spread of the data, preventing an excessively broad or overly restrictive decision boundary.

### 3.5. VAE + OCSVM

While reconstruction-based anomaly detection is effective for identifying deviations from normal sidewalk appearance, it alone cannot distinguish between anomalies that are hazardous and those that are safely traversable. In egocentric navigation, many irregular surfaces can produce large reconstruction errors, but do not pose a risk to the user. Thus, the proposed VAE + OCSVM architecture addresses this limitation by decoupling the anomaly localization (VAE) from hazard discrimination (OCSVM). This separation enables the system to generalize to unseen hazards while suppressing false alarms from non-hazardous anomalies.

This challenge arises directly from the visual variability of real-world sidewalks. Sidewalk surfaces are not consistent and frequently include elements such as electrical boxes and valve covers that are anomalous in appearance but safely traversable. When capturing data that was used to train the VAE, roughly 15% of the dataset consisted of these cases, impeding the VAE’s ability to learn meaningful features from these cases. As a result, the VAE will struggle to generate accurate reconstructions when these objects are present. These high reconstruction errors will cause the system to alert excessively, which is neither practical nor safe. Therefore, to overcome these false alerts, the results from the VAE are merged with the OCSVM.

**Figure 5:**
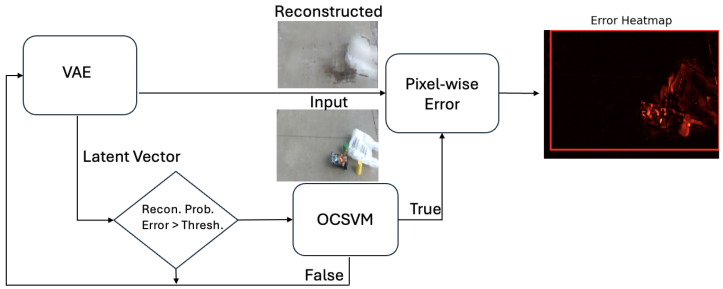
Hybrid pipeline.

When the VAE produces a −1 output (implying the presence of an anomaly), the corresponding latent vector is passed to the OCSVM. If the OCSVM also outputs −1, indicating a hazardous non-traversable anomaly, a pixel-wise mean squared error between the original input and the reconstructed output is computed to generate a heat map of the hazardous object, as illustrated in Figure 5.

## 4. Results

This section presents the experimental results of our proposed hazard detection system, detailing the structure of the training and testing datasets, the training procedure, and the model evaluation. We assess the performance of our approach using AUROC, precision–recall, and F1-score, comparing it against baseline models. Additionally, we provide an inference time analysis of detected hazards to further illustrate the system’s effectiveness for real-time applications.

### 4.1. Dataset

The dataset consists of over 20,000 unlabeled training frames depicting normal sidewalk conditions, and more than 8000 manually annotated testing frames labeled as either hazardous or non-hazardous. The dataset was collected from diverse locations, including Massachusetts, California, and Mexico City, to enhance generalization across different environments. To qualitatively assess robustness to camera variation, a small subset of images was additionally captured using a consumer smartphone (Samsung Galaxy S23, Seoul, Republic of Korea) in an egocentric configuration, focusing on the region immediately in front of the user.

Hazard annotations were performed by the data-collecting user following a predefined set of criteria. Specifically, anomalous samples were labeled as hazardous if they exhibited a significant change in elevation, surface deformation, or the presence of an object that would impede forward walking without requiring gait adaptation or detouring around the obstacle. To assess labeling consistency, a second annotator independently labeled a subset of the data. Inter-rater agreement between the two annotators was 95.4%, computed as the percentage of samples for which both annotators assigned the same label.

The training dataset includes natural variations in illumination and weather, introducing moderate shadowing and brightness variability commonly encountered during daytime outdoor navigation. However, the dataset does not include dedicated nighttime data or heavy rain scenarios, which remain challenging conditions for vision-based systems.

**Figure 6:**
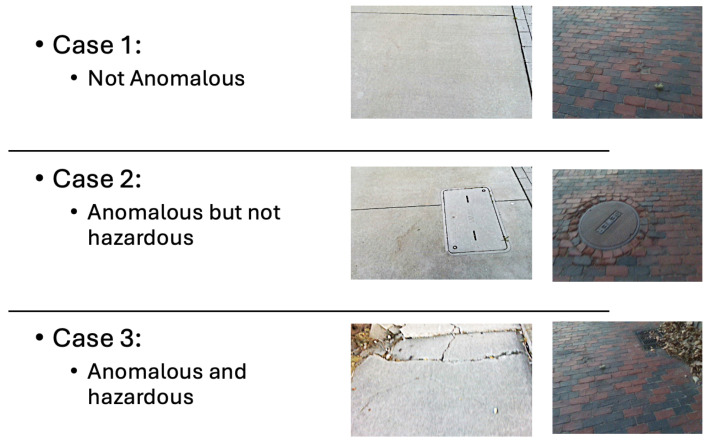
Cases of objects that can be within the dataset.

The training dataset, detailed in Table 1, was curated to ensure a diverse representation of normal sidewalk conditions, providing a robust foundation for model generalization. Within the testing dataset, there are three cases, as shown in Figure 6. Case 3 contains examples of hazardous anomalies, such as those listed in Table 2. However, the dataset is not limited to these types of anomalies, as other hazardous conditions may also be present and detectable by the system. To the best of our knowledge, this is the first dataset collected for the anomaly detection task in a sidewalk scenario.

### 4.2. Training

The VAE was trained using the training dataset for 120 epochs to learn a compact latent space representation of normal sidewalk conditions. We employed the Adam optimizer [43] with a learning rate of 1 × 10^−5^, ensuring stable convergence during training. After training, the OCSVM was trained on the latent vectors of Case 2 using a subset of the data, establishing a decision boundary that separates hazardous and non-hazardous anomalies. In this work, ν was set to 1 × 10^−3^, indicating that only 0.1% of the training data were allowed to be treated as anomalies. This small value of ν results in a tighter boundary that encloses the majority of normal (non-hazardous) data while identifying only extreme deviations as hazardous anomalies.

**Table 2:** Description of testing dataset.

Loc.	Scene	Movement	Weather	Frames	Hazard Frames	# Obj.	Description
Urban	Sidewalk	Walking	Cloudy	269	240	2	Gravel, litter
City	Sidewalk	Walking	Cloudy	96	67	1	Bottle
City	Sidewalk	Walking	Cloudy	83	53	1	Branch
City	Sidewalk	Walking	Dark	419	400	1	Water meter cover
City	Sidewalk	Walking	Clear	467	273	2	Pothole, uneven surface
Town	Sidewalk	Walking	Clear	396	214	2	Uneven surface
Town	Sidewalk	Walking	Sunny	2514	532	3	Water meter cover, terrain change, crosswalk
City	Sidewalk	Walking	Snow	246	155	2	Snow piles, ice
Town	Sidewalk	Walking	Sunny	176	176	1	Lemon
Town	Sidewalk	Walking	Sunny	1005	283	1	Loose dirt
Town	Sidewalk	Walking	Cloudy	331	141	1	Construction equipment

For baseline comparative evaluation, we trained the RealNVP and DeepSVDD models using the same encoder architecture and training parameters, ensuring a fair comparison across methods. The RealNVP model was optimized to learn an invertible transformation between input data and a simple distribution (Gaussian), allowing for exact likelihood estimation. The DeepSVDD model, on the other hand, was trained to minimize the volume of a hypersphere enclosing normal data, identifying anomalies based on their distance from this learned boundary.

The models proposed by [34,35] are pretrained architectures, which leverage large-scale datasets for classification. In our study, we incorporated these pretrained models as prior comparisons, evaluating their effectiveness in sidewalk hazard detection alongside our proposed approach.

### 4.3. Numerical Evaluation

**Figure 7:**
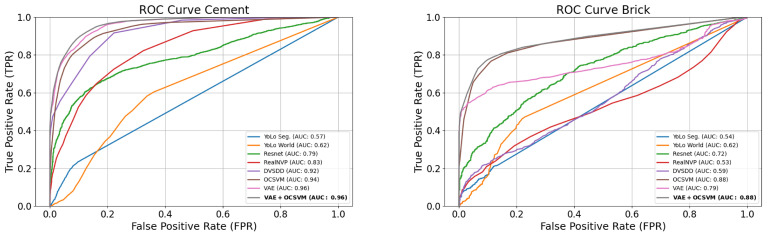
ROC curves.

**Figure 8:**
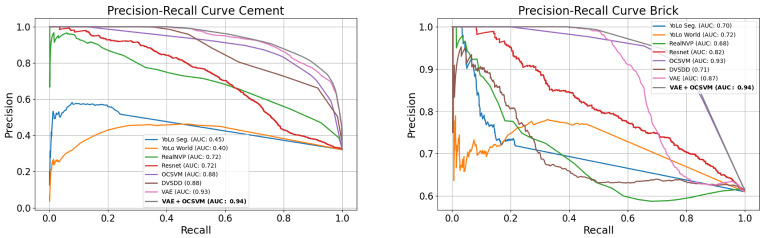
Precision Recall curves.

To evaluate the effectiveness of our proposed model, we report the Area Under the Receiver Operating Characteristic (AUROC) and Precision–Recall (PR) curve, along with the F1-score for hazardous anomaly detection. The evaluation considers two distinct sidewalk types (e.g., cement and brick), each tested under various environmental and lighting conditions across multiple locations, as shown in Table 2. Accordingly, four curves are presented: two ROC curves and two PR curves, as shown in Figure 7 and Figure 8.

Evaluation is performed using a holdout validation strategy. The dataset consists of outdoor terrain images spanning multiple semantic categories, including both anomalous and normal samples. The annotated dataset is partitioned at the category level, where a subset of anomaly categories is reserved as a validation set and used solely for selecting the decision threshold that maximizes the F1 score via grid search. This selected F1-optimal threshold is then fixed and applied to the remaining anomaly categories, which form a held-out test set, to assess generalization performance (F1-score).

We compare our framework against standard anomaly detection methods, including RealNVP and DeepSVDD. In addition, we reference other hazard detection models proposed in prior studies [34,35], which have been applied to outdoor anomaly detection scenarios. Table 3 summarizes the quantitative classification results across all methods, where standard deviations are computed across disjoint test subdatasets, with each subdataset representing a distinct anomaly type or environmental condition.

To ensure a consistent comparison across different model types, we configured the evaluation thresholds according to each model’s output characteristics. For the classification-based models proposed in prior studies [34,35], we varied the output probability in the range of [0, 1] to determine the optimal threshold for hazard detection.

Similarly, for the RealNVP model, we analyzed the likelihood-based probability scores within the same range to distinguish between normal and anomalous samples. For the DeepSVDD model, we varied the hypersphere radius threshold to separate normal data points from anomalies.

The proposed VAE + OCSVM achieved the highest averaged AUROC (0.92) and average F1-score (0.85), outperforming other methods. The individual VAE and OCSVM models performed competitively; however, their fusion further enhanced performance by leveraging the VAE’s representational capacity and the OCSVM’s discriminative boundary.

The improved performance of VAE + OCSVM can be attributed to the strong feature extraction capabilities of the VAE, allowing for a more compact and meaningful representation of the input frames. Additionally, the OCSVM effectively separates non-hazardous and hazardous anomalies, improving classification robustness. This combination enhances decision boundary precision, leading to higher recall and F1-score in anomaly hazard detection.

### 4.4. Ablation Study

To quantify the contribution of each component in the proposed framework, we evaluated (i) the VAE alone, (ii) the OCSVM alone, and (iii) the full VAE + OCSVM fusion model. As shown in the ROC and PR curves (Figure 7 and Figure 8) for both cement and brick surfaces, the VAE alone achieves strong anomaly detection performance due to its reconstruction capability, while the OCSVM alone provides a moderate boundary on latent representations. However, the fused VAE + OCSVM consistently yields the highest AUC across all conditions, improving both precision and recall. This confirms that the VAE is effective for detecting anomalous, while the OCSVM is necessary to distinguish hazardous from non-hazardous anomalies, and their integration is critical for robust hazard detection.

**Figure 9:**
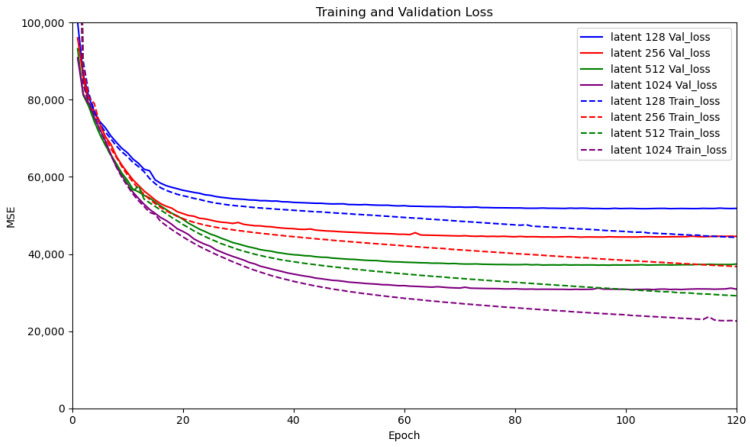
Latent Space Dimension Train and Validation curves.

To identify the latent dimensionality that minimizes the reconstruction error, we trained VAEs with latent sizes d∈{128,256,512,1024} and compared their training and validation loss curves. As shown in Figure 9, the reconstruction error decreases as the latent dimensionality increases, with d=1024 yielding the lowest validation loss. While larger latent dimensions may further reduce reconstruction error, they introduce substantially higher computational and memory costs during both training and inference. We therefore select d=1024 as a practical trade-off between reconstruction fidelity and real-time deployability. In contrast, smaller latent spaces compress the features more aggressively, resulting in consistently higher reconstruction errors.

Qualitatively, lower-dimensional latent representations produce noticeably blurrier reconstructions, which can obscure fine-grained surface irregularities. Such blurring reduces the visibility of subtle anomalies and may cause them to become indistinguishable from normal regions. Higher-dimensional latent spaces better preserve visual detail and texture variability across different sidewalk terrains, thereby providing more informative latent features for downstream anomaly classification using the OCSVM.

We also evaluated several OCSVM configurations, comparing linear and RBF kernels and testing three ν values $\left\{10^{-5},10^{-3},10^{-1}\right\}$ along with two γ strategies: γ=1/f and the median heuristic used in our final model. All results were obtained using a train/validation/test split strategy. Model configurations were trained exclusively on non-hazardous anomaly data and evaluated on the validation set to select optimal hyperparameters based on the F1 score. The final model, using the selected hyperparameters, was then evaluated on the held-out test set to report performance metrics. As shown in Table 4, the RBF kernel consistently outperformed the linear kernel, and the scale γ produced the best balance of recall and precision across all ν values. Larger ν values resulted in tight boundaries, increasing false positives, while very small ν values reduced recall. These ablations demonstrate that both the latent dimensionality and the OCSVM hyperparameters have a meaningful impact on system performance, and justify the chosen configuration used in our egocentric sensor-based hazard detection pipeline.

### 4.5. Inference Time

All networks run in real time on mobile computation hardware. Table 5 reports the inference time of anomaly detection for all models on an Macbook M2 Metal Performance Shaders with input images of 640 × 480.

## 5. Discussion

This research introduces a system that uses visual data to enhance safety navigation by detecting hazardous objects along a sidewalk. Based on the F1 score, the VAE alone achieves an accuracy of 80% using a fixed threshold. To provide context, a higher threshold increases false negatives, meaning actual hazards may be misclassified as non-hazards, potentially putting users at risk. To minimize this, a lower threshold can be used, improving hazard detection but also increasing false positives, leading to unnecessary alerts for non-hazardous objects. In some environments, elements like utility covers are common, and excessive false positives could lead to over-alerting, potentially causing alert fatigue or unnecessary panic. To mitigate this, the VAE + OCSVM hybrid model enhances classification accuracy, reducing false positives while maintaining strong performance, achieving an overall F1 score of 85%.

Because the reconstruction threshold directly influences the trade-off between false positives and false negatives, it must be calibrated for the environment in which the system is deployed. During initialization, the user stands in an obstruction-free area while the egocentric camera captures a brief sequence of frames. For each frame, the VAE reconstruction error is computed, and the operational threshold is set to the mean reconstruction error plus a small margin. This procedure adapts the system to the current lighting conditions, sensor noise, and sidewalk texture, reducing unintended anomaly detections while maintaining sensitivity to true hazards.

The VAE is used to distinguish normal sidewalk conditions from anomalies, while the OCSVM distinguishes between hazardous anomalies and non-hazardous anomalies. This method enables the VAE to be trained on extensive datasets prior to deployment, keeping the computational demands during real-time operation minimal. Moreover, given the light computational requirements and low training cost of the OCSVM, the proposed framework may support future extensions toward adaptive behavior during deployment. For instance, if a user is alerted to the same type of unrecognized anomaly but consistently navigates over it, the system could adapt by reclassifying that anomaly as non-hazardous.

While this study serves as a proof of concept for the system architecture, the data requirements for adequate training of the system for widespread deployment are not clear. Fortunately, due to the harsh winter weather in the Boston area, a wide variety of degraded sidewalk conditions are available. One challenge will be extending the system to detect elevation changes that are not visually clear. One potential solution is the incorporation of depth information from stereo cameras. This would also facilitate the detection of raised obstacles and concavities such as staircases and potholes. Depth information could be readily incorporated into the same architecture demonstrated here, albeit with increased computational demands. Additionally, environmental conditions such as strong shadows, rain, and low light illumination present challenges for vision-based anomaly detection. In our experiments, the shadowed region produced elevated reconstruction errors due to reduced texture visibility, which can negatively impact anomaly localization. While the current dataset captures moderate lighting variability, it does not explicitly model night time or heavy rain scenarios. Addressing these conditions is an important direction for future work. Potential extensions include shadow removal methods to overcome regions darkened by shadows [44]. Moreover, the Intel RealSense D435i camera includes infrared sensors that could enable operation under low-light or nighttime conditions, providing an additional sensing modality when RGB imagery becomes unreliable. In addition, future work should include a user study to evaluate the robustness of the system to variations in camera mounting height and walking speed. Such a study would help quantify how differences in user behavior and sensor placement affect anomaly detection performance and would provide valuable guidance for practical deployment across diverse users.

In addition to the results presented, a Supplementary Video S1 is available to demonstrate the implementation of this work. The Supplementary Material contains the code, video, example cases, and dataset. The video illustrates the three cases mentioned in Figure 6, providing insights into the system’s outputs along with access to the dataset and source code. The system was designed for low computational cost during deployment. The autoencoder is able to perform well with resolution images (640 × 480) and the OCSVM uses a small dimensional input as well, allowing the system to perform in real-time on a consumer laptop.

In conclusion, the key to the system’s success is the use of RGB frames in combination with machine learning [45]. The systems’ approach can increase independence when navigating environments where uncertainty is high. While this research primarily focuses on enhancing safety for the visually impaired, there are numerous potential applications beyond this scope. The system could provide crucial assistance in navigation for assistive lower-limb devices, controlling motorized wheelchairs, and legged devices, such as bipedal robots and prosthetics.

## Figures and Tables

**Table 1:** Description of training dataset.

Location	Scene	Movement	Weather	Total Images	Description
Suburbs	Sidewalk	Walking	Sunny/Cloudy	10,500	Brick
City	Sidewalk	Walking	Sunny/Cloudy	11,000	Cement

**Table 3:** Performance comparison of hazard detection models. Bold values indicate the best performance across models for each metric.

Model	AUC-ROC	AUC-PR	F1-Score
*State-of-the-Art Hazard Detection Models*			
YOLO Seg. [35]	0.55±0.01	0.58±0.34	0.33±0.35
YOLO World [35]	0.62±0.21	0.56±0.29	0.55±0.26
ResNet50 [34]	0.83±0.12	0.84±0.18	0.71±0.17
*Baseline Models*			
RealNVP	0.68±0.14	0.70±0.17	0.64±0.16
Deep SVDD	0.76±0.21	0.80±0.22	0.77±0.16
OCSVM	0.91 ± 0.08	0.91 ± 0.08	0.78 ± 0.10
VAE	0.88 ± 0.05	0.90 ± 0.03	0.80 ± 0.07
*Ours*			
**VAE + OCSVM**	**0.92 ± 0.04**	**0.94 ± 0.03**	**0.82 ± 0.06**

**Table 4:** OCSVM Ablation Study.

Kernel	ν	Strategy (γ)	Recall	Precision	F1
rbf	1 × 10^−3^	scale	0.90	0.96	0.93
rbf	1 × 10^−1^	median	0.89	0.96	0.92
rbf	1 × 10^−1^	scale	0.93	0.92	0.92
linear	1 × 10^−5^	–	0.58	0.45	0.50
linear	1 × 10^−1^	–	0.47	0.40	0.43
linear	1 × 10^−3^	–	0.43	0.36	0.39

**Table 5:** Runtime comparison of hazard detection models.

Model	Time [ms]	Rate [FPS]
*State of the Art Hazard Detection Models*		
YoLo Seg. [35]	1377.6	0.73
YoLo World [35]	1464.8	0.68
ResNet50 [34]	24.71	40.47
*Baseline Models*		
RealNVP	22.82	43.82
Deep SVDD	6.48	154.22
OCSVM	6.48	154.22
VAE	22.01	45.43
*Ours*		
VAE + OCSVM	25.45	39.29

## Data Availability

The dataset generated and analyzed in this study is available on Harvard Dataverse at https://doi.org/10.7910/DVN/ZLYKI9. All code used for training, evaluation, and anomaly detection experiments is publicly available as described in the Supplementary Materials.

## Supplementary Materials

The following supporting information can be downloaded at: https://github.com/erguzman0808/Sidewalk-Hazard-Detection-Using-a-Variational-Autoencoder-and-One-Class-SVM (accessed on 18 January 2026). Supplementary Code: Python implementation of the variational autoencoder (VAE), one-class SVM (OCSVM), training scripts, ROC and precision–recall curve evaluation scripts. Video S1: Demonstration of hazard detection results using the proposed VAE + OCSVM pipeline on outdoor sidewalk scenes.
